# Predictors of Surgical Complications and Survival in Pediatric Wilms’ Tumor: A 20-Year Retrospective Study from Two Thai Centers

**DOI:** 10.3390/curroncol32080413

**Published:** 2025-07-23

**Authors:** Wison Laochareonsuk, Mongkol Laohapansang, Monawat Ngerncham, Surasak Sangkhathat

**Affiliations:** 1 Division of Pediatric Surgery, Department of Surgery, Faculty of Medicine, Siriraj Hospital, Mahidol University, Bangkok 10700, Thailand; wison.l@psu.ac.th (W.L.); mongkol.lao@mahidol.ac.th (M.L.); 2 Division of Pediatric Surgery, Department of Surgery, Faculty of Medicine, Prince of Songkla University, Songkhla 90110, Thailand; 3 Translational Medicine Research Center, Faculty of Medicine, Prince of Songkla University, Songkhla 90110, Thailand

**Keywords:** Wilms’ tumor, nephroblastoma, surgical complications, neoadjuvant chemotherapy

## Abstract

(1) Background: Wilms’ tumor (WT) is the most common pediatric renal malignancy. Although survival outcomes have improved with multimodal therapy, the optimal sequence of surgery and chemotherapy remains debated, particularly in resource-limited settings. This study evaluates the effect of treatment strategy on surgical complications and survival, utilizing two decades of data from Thai tertiary centers. (2) Methods: A retrospective cohort study was conducted on 83 children who underwent radical nephrectomy for WT between 2002 and 2022 at two university hospitals in Thailand. Patients were grouped by treatment protocol: primary nephrectomy (n = 59) or neoadjuvant chemotherapy (n = 24). Clinical, pathological, operative, and follow-up data were analyzed to identify predictors of surgical complications and survival. (3) Results: Short-term postoperative complications occurred in 16.9% of cases, more frequently in males and in patients with hypoalbuminemia, anemia, or large tumors. Estimated blood loss greater than 5 mL/kg, serum albumin less than 3.5 g/dL, and hemoglobin lower than 10 g/dL were independent predictors of complications. The five-year overall survival (OS) and progression-free survival (PFS) rates were 82.4% and 68.1%, respectively. Patients with unfavorable histology or short-term complications experienced significantly poorer OS. Neoadjuvant chemotherapy was associated with increased nutritional compromise and a trend toward higher complication rates, although it was not directly linked to inferior OS. (4) Conclusions: In pediatric WT, the perioperative nutritional and hematologic statuses significantly influence surgical outcomes. While neoadjuvant chemotherapy may assist in tumor downsizing, it might also compromise surgical fitness. Customized preoperative risk assessment and nutritional support can enhance outcomes, particularly in low- and middle-income countries.

## Simple Summary

This 20-year retrospective study of 83 Thai children with Wilms’ tumor compared outcomes between primary nephrectomy and neoadjuvant chemotherapy. Postoperative complications occurred in 16.9%, mainly linked to poor nutritional and hematologic status (low albumin, anemia, high blood loss). Five-year overall survival was 82.4%, with worse outcomes seen in patients with unfavorable histology or complications. Neoadjuvant chemotherapy helped reduce tumor size but worsened nutritional status, potentially raising complication risks without significantly impacting survival. Optimizing nutrition and preoperative risk assessment may improve outcomes, especially in resource-limited settings.

## 1. Introduction

Wilms’ tumor (WT), or nephroblastoma, is the most common malignant renal neoplasm in children, accounting for approximately 90% of pediatric kidney cancers [1,2]. It typically presents in children under five years of age, with an estimated incidence of 1 in 10,000 [3]. Histologically, WT arises from metanephric blastema and is characterized by a triphasic pattern comprising blastemal, epithelial, and stromal components [4]. The pathogenesis is believed to follow a “two-hit” model involving mutations in key developmental genes, most notably *WT1* on chromosome 11p13, and aberrations at 11p15, which are also implicated in overgrowth syndromes such as Beckwith–Wiedemann and WAGR [5]. Over the past few decades, multimodal treatment strategies, incorporating surgery, chemotherapy, and radiotherapy, have dramatically improved survival outcomes, with overall survival (OS) rates exceeding 90% for localized disease and 70–80% for metastatic cases [6,7,8]. Research is underway on molecular targeted therapy and the optimization of the treatment protocols [9].

The timing of surgical intervention remains a topic of clinical debate. While the Children’s Oncology Group (COG) protocol advocates for upfront nephrectomy followed by adjuvant therapy, the International Society of Paediatric Oncology (SIOP) recommends preoperative chemotherapy to reduce tumor volume and surgical risks [10,11]. Although both strategies have demonstrated comparable survival outcomes in large trials, their respective impacts on perioperative complications and long-term sequelae in real-world settings remain underexplored, particularly in low- and middle-income countries (LMICs), where resource constraints may influence treatment sequencing and patient optimization [7,8,12]. The choice between these treatment approaches could significantly affect patient management and outcomes, highlighting the need for further research in diverse healthcare settings [13]. A previous study at Songklanagarind Hospital, a tertiary care center in southern Thailand, reported that WT with a large tumor size at presentation (>10 cm) was associated with a higher risk of poorer survival outcomes [13]. The study found that a large tumor poses more risk of primary surgical failure and increases the risk of early recurrence. For this reason, surgical decisions at the institute tended to adopt an upfront chemotherapy scheme for cases with a large tumor volume at presentation. A follow-up study focusing on radiological features also demonstrated correlations between major vascular encasement, adrenal involvement, and intraoperative tumor spillage, and higher operative risk and decreased chance of survival [10]. However, chemotherapy may hamper the patient’s general condition, making them less suited for surgery.

Emerging data suggest that certain preoperative clinical factors—such as nutritional status, tumor burden, and hematologic parameters—may predispose patients to postoperative complications and affect long-term survival [10,11,12]. However, few studies have systematically examined these predictors in WT patients across diverse treatment strategies. The question raised is whether, when surgical techniques and perioperative care improve, upfront chemotherapy is still a good option for WT with anatomical risks.

This retrospective study analyzes 20 years of surgical experience at two major pediatric surgical centers in Thailand. We aimed to compare clinical outcomes between children receiving primary nephrectomy and those undergoing neoadjuvant chemotherapy. The second objective was to identify predictors of surgical complications and survival. By examining both treatment protocols within a single national context, our study offers practical insights into optimizing perioperative care and long-term outcomes in pediatric WT.

## 2. Materials and Methods

### 2.1. Study Design and Setting

We conducted a retrospective cohort study involving pediatric patients diagnosed with WT who underwent radical nephrectomy at two tertiary-care centers in Thailand: Siriraj Hospital, Mahidol University (Bangkok), and Songklanagarind Hospital, Prince of Songkla University (Songkhla). The study period spanned from January 2002 to December 2022. Institutional review board approval was obtained from both participating institutions prior to data collection (HREC.REC.65-473-10-1 and IRB1.915/2565).

### 2.2. Patient Eligibility

The inclusion criteria were as follows: (1) age < 18 years at diagnosis, (2) histologically confirmed Wilms’ tumor (nephroblastoma), and (3) definitive surgical management via radical nephrectomy. Patients were categorized based on initial treatment approach: either primary nephrectomy or neoadjuvant chemotherapy followed by delayed surgery. Treatment regimens adhered to either the Children’s Oncology Group (COG) or the International Society of Paediatric Oncology (SIOP) protocols, depending on institutional preference and disease presentation (Supplementary Tables S3 and S4). Evaluation of effectiveness of preoperative chemotherapy occurred after six weeks of chemotherapy. Postoperative chemotherapy and radiation therapy followed the Thai Pediatric Oncology Group’s protocol (TPOG 2016) [13] (Supplementary Tables S5 and S6).

### 2.3. Data Collection

De-identified data were extracted from electronic and physical medical records using a standardized collection form. Demographic variables included age at diagnosis, sex, body weight, and presence of associated syndromes or congenital anomalies. Clinical variables included presenting symptoms, blood pressure at diagnosis, laboratory findings (complete blood count, serum biochemistry, and urinalysis), and radiologic tumor characteristics. The unfavorable histology criteria included a tumor with anaplastic features. For SIOP histopathological risk group classification, the tumors were categorized by their pathology as low, intermediate, or high risk [14].

The operative details recorded were duration of surgery, estimated blood loss (EBL), surgical resection status, and intraoperative complications. Tumor characteristics—such as laterality, size, weight, histological subtype, and staging—were obtained from surgical and pathology reports. Postoperative data included time to oral intake, duration of hospitalization, and incidence of short- and long-term complications. Complications were predefined and categorized according to type and timing relative to surgery. A short-term complication was defined as an event that occurred within 30 days postoperatively. All patients were followed for at least 5 years postoperatively or until death.

### 2.4. Outcome Measures

The primary outcomes were as follows:

1. Incidence and types of postoperative complications, both short-term (less than 30 days) and long-term (more than 30 days).
2. Indicators of complications based on preoperative clinical, laboratory, and tumor-related factors.
3. Overall survival (OS) and progress-free survival (PFS) are defined as the time from diagnosis to death from any cause and to the first occurrence of relapse, progression, new metastasis, or death, respectively.

### 2.5. Statistical Analysis

Continuous variables were expressed as mean ± standard deviation and compared using Student’s *t*-test or the Mann–Whitney U test, as appropriate. Categorical variables were compared using the Chi-square test or Fisher’s exact test, where appropriate. Multivariable logistic regression was used to identify independent predictors of surgical complications. Median follow-up time was up to the last follow-up visit or, in cases of death, December 2024. Survival outcomes were analyzed using Kaplan–Meier curves and compared using the log-rank test. The Cox proportional-hazards model was applied to assess factors associated with OS and PFS. A *p*-value < 0.05 was considered statistically significant. Analyses were performed using STATA version 14.0 (StataCorp LLC, College Station, TX, USA).

## 3. Results

### 3.1. Patient Characteristics

A total of 83 children with Wilms’ tumor underwent radical nephrectomy during the study period. Of these, 59 patients (71%) received primary nephrectomy, and 24 (29%) underwent preoperative chemotherapy. Baseline demographics, clinical presentation, and preoperative laboratory values are summarized in Table 1.

The mean age at surgery was 2.7 ± 1.9 years in the primary surgery group and 2.3 ± 1.6 years in the neoadjuvant group (*p*-value = 0.318). A palpable abdominal mass was the most frequent presenting symptom (66%), followed by hypertension (55%). Congenital anomalies were documented in eleven patients, with WAGR syndrome (OMIM 194072) in eight (9.6%) and horseshoe kidney in three (3.6%).

Of 40 cases operated on in Siriraj Hospital, 2 cases (5%) received neoadjuvant chemotherapy, whereas 22/43 cases (55%) operated on in Songklanagarind Hospital did so (*p*-value < 0.01). Preoperative chemotherapy was associated with significantly lower white blood cell (WBC) and absolute neutrophil counts (ANC) (*p*-values = 0.015 and 0.003, respectively). No differences in serum albumin or hemoglobin levels were observed between groups. Renal function markers (blood urea nitrogen and creatinine) were lower in the neoadjuvant group (*p*-value < 0.05), likely reflecting better preoperative optimization.

### 3.2. Tumor Characteristics

Postoperative staging revealed that Stage III was the most frequently observed tumor stage, occurring in 39.0% of patients in the primary nephrectomy group and 45.8% in the preoperative chemotherapy group. A statistically significant difference in overall stage distribution was identified between the two treatment groups (*p* = 0.004; Table 1). Almost all Stage I patients (12/13 cases) underwent a primary nephrectomy, whereas 6/7 of bilateral WT (Stage V) had upfront chemotherapy. Tumors were more frequently localized in the left kidney (61.5% of cases), with no significant predominance of laterality by treatment group. Distant metastasis was identified in seven patients (11.9%) in the primary nephrectomy group and one patient (4.2%) in the neoadjuvant group. Among these, the lungs were the most common metastatic site, involved in six cases (7.2%).

Radiographic imaging at diagnosis estimated the mean tumor size at 9.68 ± 2.23 cm, which corresponded closely with the mean pathological tumor size of 9.62 ± 2.69 cm. A statistically significant difference was observed between radiologic and histologic measurements across both treatment groups (*p* < 0.001). Mean tumor weight was 584.4 ± 336.0 g, and mean tumor volume was 1037.8 ± 864.6 cm^3^; these metrics did not differ significantly by treatment sequence.

Favorable histology was the predominant subtype, present in 88.0% of the cohort. We found that cases in the primary nephrectomy group had a significantly higher proportion of intermediate to high SIOP histopathology. Renal capsule invasion occurred more frequently in the primary nephrectomy group (37.3%) compared to the neoadjuvant group (8.3%), a statistically significant difference (*p* = 0.008). Lymphovascular invasion was noted in 42.2% of tumors, without significant variation between groups (*p* = 0.299).

Most patients had negative para-aortic lymph nodes, including 84.8% in the primary surgery group and 95.8% in the neoadjuvant group. Tumor thrombus was observed in 9.0% of patients, and tumor rupture prior to surgery was documented in 11.1% of cases. Negative surgical margins were achieved in over 90% of both groups, with slightly more in the neoadjuvant chemotherapy group (Table 2).

### 3.3. Operations and Postoperative Follow-Up

Radical nephrectomies were performed by experienced pediatric surgical oncology teams at both institutions. As summarized in Table 2, there was no statistically significant difference in operative time between the primary nephrectomy group (mean 290.5 ± 118.0 min) and the preoperative chemotherapy group (mean 274.9 ± 65.3 min; *p*-value = 0.543). Estimated intraoperative blood loss was also comparable between groups (140.9 ± 230.1 mL vs. 217.4 ± 506.0 mL; *p*-value = 0.344). Complete tumor resection was achieved in 72 out of 83 patients (86.8%), with no significant difference in resection status between treatment groups.

Postoperatively, the average duration of enteral diet withholding was 2.38 ± 1.49 days, and the mean time to full oral intake was 4.01 ± 2.91 days (Table 3). No significant differences were noted between groups in nil-per-oral duration (*p*-value = 0.897) or time to full feeding (*p*-value = 0.212). The average length of postoperative hospital stay was 16.1 ± 12.9 days for the primary surgery group and 12.8 ± 9.5 days for the neoadjuvant chemotherapy group, with no statistically significant difference observed (*p*-value = 0.249).

All patients received adjuvant chemotherapy following tumor resection. However, a significantly greater proportion of patients in the preoperative chemotherapy group received adjuvant radiotherapy, compared to the primary nephrectomy group (75.0% vs. 47.5%, *p*-value = 0.022). During the follow-up period, tumor recurrence was observed in 24.1% of patients, with a higher percentage of recurrence in the preoperative chemotherapy group. However, the difference did not reach a statistically significant level. Overall mortality was 16.9%, with a higher percentage, but not significantly higher, in the neoadjuvant group (Table 3).

### 3.4. Short-Term and Long-Term Postoperative Complications

As the second focus of our study was on operative complications, we reviewed all details of postoperative events. Short-term postoperative complications were documented in 19 patients—10 from the primary excision group and 9 from the preoperative chemotherapy group—as detailed in Table 4. A statistically significant difference was observed in the incidence of urinary tract infections and septicemia, with a higher occurrence in the preoperative chemotherapy group. Regarding long-term postoperative complications, bowel obstruction was reported in seven patients (8.43%), while incisional hernia was noted in one patient (1.20%). Additionally, chronic kidney disease was diagnosed in four patients (4.82%) during the long-term follow-up phase.

### 3.5. Correlation Between Complications and Surgery-Related Factors

Factors analyzed for their association with operative complications were categorized into patient factors, operative factors, and tumor factors.

### 3.6. Patient Factors

Male patients experienced a higher incidence of short-term postoperative complications than their female counterparts, with 78.57% (eleven cases) of complications occurring in males and 21.43% (three cases) in females, as shown in Supplementary Tables S1 and S2. Analysis of preoperative hemoglobin levels revealed a significantly lower value in patients who later developed complications (mean 9.52 ± 2.15 g/dL) compared to those without complications (mean 10.64 ± 1.64 g/dL), *p* = 0.031. Furthermore, a notable difference was observed in preoperative serum albumin levels, with cases exhibiting no complications averaging 4.07±0.49 g/dL, while complicated cases had a lower mean of 3.67 ± 0.53 g/dL, a discrepancy that holds statistical significance (*p* = 0.009). 

### 3.7. Operative Factors

The duration of surgery did not differ significantly between groups (*p* = 0.224). Similarly, the estimated blood loss was comparable across groups (*p* = 0.291). Within the complication cohort, there was a higher incidence of positive surgical margins (21.43% compared to 5.8%); however, this difference was not statistically significant.

### 3.8. Tumor Factors

The primary tumor site was predominantly observed as being on the left side, with left-sided WT registering significantly more short-term postoperative complications (12 cases, 85.71%) compared to right-sided ones (*p* = 0.041), as detailed in Table S1. Statistical analysis revealed disparities in tumor stage distribution between patients with postoperative complications and those without, with the former group exhibiting a tendency towards more advanced tumor stages and a greater incidence of bilateral tumors. Tumor size was not significantly linked to the development of complications; however, an increase in tumor weight and volume was found to be a statistically significant predictor of short-term complications. No statistical correlations were found between other indicators of tumor aggressiveness, such as histologic subtype, lymphovascular invasion, local invasion, tumor rupture, and lymph node metastasis, and the incidence of postoperative complications.

Patients experiencing short-term postoperative complications demonstrated a significant delay in resuming oral intake (NPO time) and in progressing to full feeding (*p* = 0.003 and *p* = 0.001, respectively), resulting in hospital stays being prolonged by approximately 10 days, as presented in Table 4. Logistic regression analysis identified an estimated blood loss exceeding 5 mL/kg as a prominent predictor for the onset of short-term complications (*p* = 0.015). Additionally, patients presenting with serum albumin levels below 3.5 g/dL and hemoglobin levels below 10 g/dL were found to have an increased risk of developing postoperative complications.

Serum albumin levels were identified as a significant prognostic indicator for the occurrence of both short-term and long-term postoperative complications (Table 4). Additionally, the presence of hypertension at initial presentation was significantly associated with an increased risk of developing long-term complications.

### 3.9. Survival Outcomes

**Figure 1:**
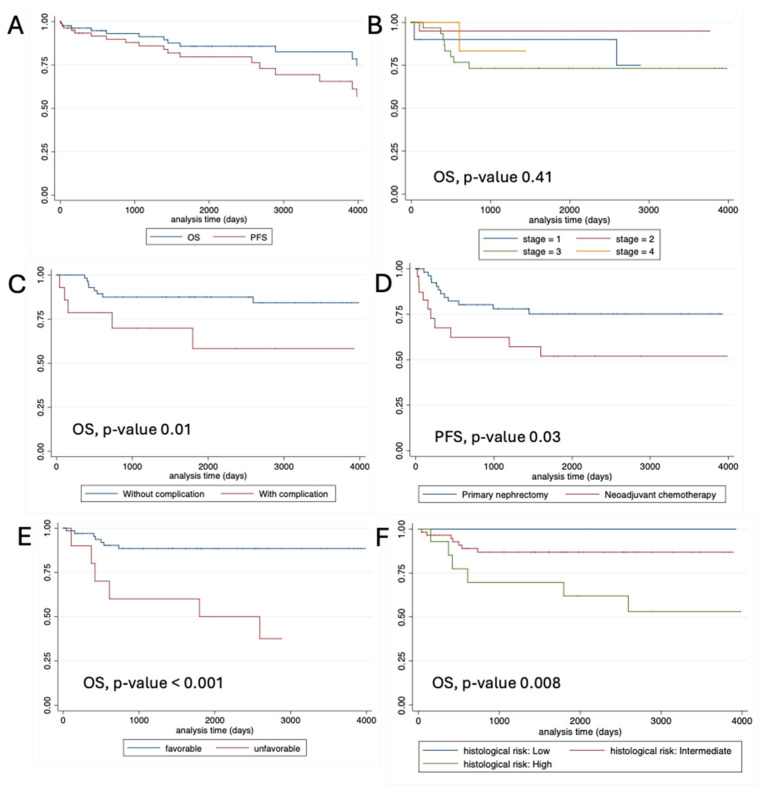
Kaplan–Meier survival curves (**A**): demonstrating overall survival (OS) and progress-free survival; (**B**): comparing OS among stages I to IV; (**C**): comparing OS between those with and without short-term postoperative complications; (**D**): comparing PFS between those who underwent a primary nephrectomy and those who received neoadjuvant chemotherapy; (**E**): comparing OS probability between Wilms’ tumor with favorable and unfavorable histology; and (**F**): comparing OS probability among Wilms’ tumor with SIOP’s low, intermediate, and high histology risks. All comparisons (**B**–**F**) used the log-rank test.

The median follow-up period was 70.5 months. After a minimum follow-up of five years, the 2- and 5-year OS rates for the entire cohort were 85.9% and 82.4%, respectively. PFS rates at 2 years and 5 years were 75.0% and 68.1%, respectively (Figure 1). Patients with unfavorable histology and/or positive resection margins had significantly poorer OS and PFS. Cox proportional-hazards modeling revealed that unfavorable histology was associated with a hazard ratio (HR) of 6.14 for OS (95% CI: 2.06–18.29). The SIOP histologic risk classification was significantly associated with OS (HR 4.30, 95% CI: 1.57–11.8) and PFS (HR 2.53, 95% CI: 1.12–5.74). The presence of a short-term complication was linked to an HR 3.61 for OS (95% CI: 1.25–10.47, *p*-value = 0.026). Interestingly, the presence of short-term complications was associated with poorer OS (HR3.61, 95% CI: 1.25–10.47). The treatment protocol was significantly associated with PFS (HR 2.50, 95% CI: 1.08–5.80), but not OS. A log-rank test for complication predictors and survival outcomes is presented in Table 5.

## 4. Discussion

Modern multidisciplinary management for pediatric WT has substantially improved outcomes associated with the disease. Compared with our previous report in 2008, the all-stage 5-year OS of 82.4% is much improved from the 65% in that year [15]. This study not only determines the current outcome figures for WT from large university centers in Thailand but also tries to answer a question as to the sequence of treatment that would be suitable for a resource-limited setting in that country. During the study period, in 2016, the Thai Pediatric Oncology Group launched a National Protocol for the treatment of childhood cancers, which included a guideline for WT management [13]. The protocol provides various postoperative chemotherapy regimens for WT according to stages and histologic subtypes. However, there was no consensus on the timing of surgery, neoadjuvant therapy, or a specific protocol for unresectable cases. In general, primary nephrectomy is advised unless the primary tumor is deemed unresectable. However, from the surgical point of view, assessment of surgical risk varies among different surgical settings. With prior data showing that intraoperative rupture of large WT led to early recurrence, which hampered survival outcomes, surgeons in the second institute in this study (PSU) preferred not to perform a primary nephrectomy in most WT with an initially large tumor size (more than 10 cm at its greatest diameter), while the other institution (Siriraj) preferred to adhere to the COG protocol. This gave an opportunity to analyze the differences in surgical and survival outcomes.

Our data showed that primary nephrectomy fared better in terms of patients’ general health before surgery. The neoadjuvant chemotherapy group had a significantly higher incidence of anemia and hypoalbuminemia, indicating that chemotherapy may have consequences on nutritional status and/or bone marrow function. Although the study did not demonstrate a direct association between the treatment protocol and surgical complications, the analysis revealed a significant influence of anemia and/or hypoalbuminemia on the likelihood of postoperative events, especially infectious complications. Furthermore, the presence of short-term complications significantly jeopardized progression-free survival. One limitation of this interpretation lies in the finding that those who experienced complications also had significantly larger tumor weights, which may suggest that larger tumor size influences the risk of complications. This evidence is consistent with a cohort that reported a significant association between a tumor volume of greater than 1000 mL and increased risk of intraoperative spill [16]. A recent study also stated that WT with anatomical risks, especially vascular encasement and the presence of preoperative tumor spillage, was associated with a higher risk of having surgical complications [10]. In that study, tumor size was not associated with any difference between high and low surgical risks. In another study, the relative tumor area compared to the abdominal area of the same image slice was described as an indicator of tumor spill risk. Taken together, this could imply that tumor size does matter. However, stratifying risk for preoperative chemotherapy should not solely rely on size, but consider the extent of extracapsular disease, and associated anatomical anomalies such as a horseshoe kidney. Bilateral WT, which deserves a nephron-sparing surgery, is another condition necessitating preoperative chemotherapy. The presence of a distant metastasis is one of the indications for upfront chemotherapy. However, in a resource-limited setting, metastatic work-up might take longer than surgery. This was the reason that most of our Stage IV cases underwent a primary nephrectomy before adjuvant treatment.

The data also suggested that nutrition and hematological status influence operative outcomes. Cases with preoperative visceral protein deficiency and anemia were at risk of developing immediate operative complications, and these conditions were more likely to be found in cases with neoadjuvant treatment. A study from an LMIC estimated that 66% of children with WT had malnutrition, as determined by serum albumin and arm anthropometry [17]. Recent studies have emphasized the importance of perioperative nutritional management in improving outcomes in WT patients [18,19].

Adjuvant treatment in the TPOG protocol did not consider response to chemotherapy or the presence of blastemal histology as risk factors. However, when we retrospectively analyzed the correlation between the SIOP histopathological risk classification and survival outcomes, the high-risk group was significantly linked to poorer survival. We also found that failure in our Stage I case occurred in a patient with the blastemal subtype, which was deemed high risk by SIOP histopathology. This case underwent a nephrectomy, followed by a course of standard chemotherapy without radiation therapy. The recurrence happened in the fourth year of follow-up. The evidence suggests that histopathologic risk should also be taken into account when choosing the treatment protocol.

Our study has limitations due to its retrospective nature, which precludes randomization between groups. However, with the assumption that both institutions provided a comparable standard of care, the analysis provides insightful data on outcome comparison between the two treatment schemes. Another limitation was that the pathological risk classification was retrospectively reviewed later, without being incorporated into the treatment scheme. In addition, delays in staging might be a cause of deviation from an accurate postoperative adjuvant in a number of cases.

In conclusion, the study retrospectively analyzed the surgical outcomes of WT using collaborative data from two university hospitals in Thailand. The data suggested that the survival of localized WT can be expected at a rate over 90%, while locally advanced and metastatic WT still had a relatively high chance of failure due to recurrence and the progression of disease. Neoadjuvant therapy was indirectly associated with a higher chance of operative complications by jeopardizing the general status of the patients. Further research should focus on selecting cases that would have benefited from upfront chemotherapy. Additionally, risk reduction through perioperative nutritional support may help improve outcomes.

## Figures and Tables

**Table 1:** Baseline demographic and clinical characteristics of pediatric patients diagnosed with Wilms’ tumor at two tertiary centers in Thailand, 2003–2022. (Values are presented as number (%) for categorical variables and mean (standard deviation) for continuous variables, unless otherwise specified.)

Variable	Primary Nephrectomy (n = 59)	Neoadjuvant Chemo (n = 24)	Total (n = 83)	*p*-Value
**Demographics**				
Age at surgery (days)	1008.5 ± 693.9	846.2 ± 594.5	961.0 ± 667.3	0.318
Male sex	30 (50.8)	14 (58.3)	44 (53.0)	0.536
Body weight (kg)	12.66 ± 4.10	11.45 ± 3.82	12.31 ± 4.03	0.217
**Clinical Presentation**				
Abdominal mass	38 (64.4)	17 (70.8)	55 (66.3)	0.575
Hypertension	28 (47.5)	18 (75.0)	46 (55.4)	**0.022**
**Associated Anomalies**				
WAGR syndrome	7 (11.9)	1 (4.2)	8 (9.6)	0.281
Horseshoe kidney	3 (100.0)	0 (0.0)	3 (3.6)	0.260
**Preoperative Lab Values**				
Hemoglobin (g/dL)	10.36 ± 1.81	10.69 ± 1.68	10.46 ± 1.77	0.447
WBC (×10^3^/μL)	12.39 ± 4.33	10.01 ± 2.67	11.70 ± 4.05	**0.015**
ANC (×10^3^/μL)	6.68 ± 4.04	4.00 ± 2.15	5.91 ± 3.78	**0.003**
Serum albumin (g/dL)	3.98 ± 0.52	4.07 ± 0.52	4.01 ± 0.52	0.490
Creatinine (mg/dL)	0.40 ± 0.15	0.31 ± 0.12	0.38 ± 0.15	**0.012**
**Postoperative surgical staging**				
Stage I	12 (20.34)	1 (4.17)	13 (15.66)	**0.004** ^α^
Stage II	16 (27.12)	5 (20.83)	21 (25.30)	
Stage III	23 (38.98)	11 (45.83)	34 (40.96)	
Stage IV	7 (11.86)	1 (4.17)	8 (9.64)	
Stage V	1 (1.69)	6 (25.00)	7 (8.43)	

Statistical comparisons were made using the Chi-square test or Fisher’s exact test for categorical variables and the Mann–Whitney U test for continuous variables; Bold *p*-values indicate statistical significance (*p*-value < 0.05); WAGR syndrome: Wilms’ tumor, Aniridia, Genitourinary tract anomalies, Renal anomalies syndrome; WBC: White blood cell count; ANC: Absolute neutrophil count.

**Table 2:** Tumor characteristics, operative variables, and postoperative outcomes for pediatric Wilms’ tumor patients undergoing primary nephrectomy or neoadjuvant chemotherapy.

Variable	Primary Nephrectomy (n = 59)	Neoadjuvant Chemo (n = 24)	Total (n = 83)	*p*-Value
**Operative Variables**				
Operative time (min)	290.5 ± 118.0	274.9 ± 65.3	286.0 ± 105.3	0.543
Estimated blood loss (mL)	140.9 ± 230.1	217.4 ± 506.0	163.0 ± 332.4	0.344
Complete gross resection	51 (86.4)	21 (87.5)	72 (86.8)	0.897
**Laterality of primary tumor**				
Left	36 (61.62)	10 (41.67)	51 (61.45)	**0.002 ^α^**
Right	22 (37.29)	8 (33.33)	32 (38.55)	
Bilateral	1 (14.29)	6 (25.00)		
**Tumor size (cm)**	9.84 (2.19)	9.09 (3.64)	9.62 (2.69)	0.254 ^β^
**Tumor weight (gm)**	579.15 (295.67)	599.63 (441.96)	584.41 (336.02)	0.821 ^β^
**Tumor volume (cm^3^)**	1029.68 (617.71)	1057.87 (1131.762)	1037.83 (864.61)	0.884 ^β^
**Local invasion**				
Renal capsule	22 (37.29)	2 (8.33)	24 (28.92)	**0.008** ^α^
Renal sinus	16 (27.12)	2 (8.33)	18 (21.69)	0.060 ^α^
Renal vein	7 (11.86)	1 (4.17)	8 (9.64)	0.281 ^α^
Gerota’s fascia	5 (8.47)	0 (0)	5 (6.02)	0.141 ^α^
Adjacent organs	4 (6.78)	0 (0)	4 (4.82)	0.191 ^α^
**Site of distant metastasis**				
Lung	6 (10.17)	0 (0)	6 (7.23)	0.105 ^α^
Liver	1 (1.69)	0 (0)	1 (1.20)	0.521 ^α^
Bone	1 (1.69)	1 (4.17)	2 (2.41)	0.506 ^α^
**Tumor rupture**	7 (12.28)	2 (8.33)	9 (11.11)	0.606 ^α^
**Tumor thrombus**	4 (7.02)	3 (14.29)	7 (8.97)	0.319 ^α^
**Paraaortic lymph node**				
Negative	50 (84.75)	23 (95.83)	73 (87.95)	0.159 ^α^
Positive	9 (15.25)	1 (4.17)	10 (12.05)	
**Ascites**	7 (12.50)	0 (0)	7 (8.75)	0.070 ^α^
**Histologic margin status**				
Negative	53 (89.83)	23 (95.83)	76 (91.57)	0.372 ^α^
Positive	6 (10.17)	1 (4.17)	7 (8.43)	
**Histologic subtype**				
Favorable histology	52 (88.14)	21 (87.50)	73 (87.95)	0.936 ^α^
Unfavorable histology	7 (11.86)	3 (12.50)	10 (12.05)	
**SIOP histopathological risk**				
Low risk	0 (0)	4 (16.66)	4 (4.82)	**0.003**
Intermediate risk	52 (88.14)	13 (54.17)	65 (78.31)	
High risk	7 (11.86)	7 (29.17)	14 (16.87)	
**Lymphovascular invasion**	27 (45.76)	8 (33.33)	35 (42.17)	0.299 ^α^

Data are presented as mean ± SD for continuous variables and number (percentage) for categorical variables. Significant *p*-values (<0.05) are indicated in bold. Statistical comparisons were made using Fisher’s exact test (α) or independent *t*-test (β), as appropriate.

**Table 3:** Postoperative outcomes of pediatric Wilms’ tumor patients undergoing primary nephrectomy or neoadjuvant chemotherapy.

Variable	Primary Nephrectomy (n = 59)	Neoadjuvant Chemo (n = 24)	Total (n = 83)	*p*-Value
**Time to initial feeding (days)**	2.25 (1.22)	2.70 (2.01)	2.38 (1.49)	*0.212 ^β^*
**Time to full feeding (days)**	4.06 (3.16)	3.87 (2.23)	4.01 (2.91)	*0.786 ^β^*
**Length of hospital stay (days)**	16.13 (12.91)	12.75 (9.52)	15.15 (12.07)	*0.249 ^β^*
**Post-op adjuvant therapy**				
Chemotherapy	59 (100)	24 (100)	83 (100)	*-*
Radiotherapy	28 (47.46)	18 (75.00)	46 (55.42)	***0.022*** *^α^*
**Short-term complications**				
Vascular injury	2 (3.39)	0 (0)	2 (2.41)	*0.361 ^α^*
Intraabdominal hemorrhage	1 (1.69)	0 (0)	1 (1.20)	*0.521 ^α^*
Diaphragm injury	3 (5.08)	1 (4.17)	4 (4.82)	*0.859 ^α^*
Chyle leakage	1 (1.69)	1 (4.17)	2 (2.41)	*0.506 ^α^*
Pneumonia	2 (3.39)	1 (4.17)	3 (3.61)	*0.864 ^α^*
Urinary tract infection	0 (0)	2 (8.33)	2 (2.41)	***0.025*** *^α^*
Sepsis	1 (1.69)	4 (16.67)	5 (6.02)	***0.009*** *^α^*
**Long-term complications**				
Bowel obstruction	6 (10.17)	2 (4.17)	7 (8.43)	*0.372 ^α^*
Incisional hernia	0 (0)	1 (4.17)	1 (1.20)	*0.115 ^α^*
Chronic kidney disease	2 (3.39)	2 (8.33)	4 (4.82)	*0.340 ^α^*
**Disease status**				
Progress-free	48 (81.36)	15 (62.50)	63 (75.90)	*0.069 ^α^*
Recurrence	11 (18.64)	9 (37.50)	20 (24.10)	
**Patient status**				
Alive	51 (86.44)	18 (75.00)	69 (83.13)	*0.207 ^α^*
Death	8 (13.56)	6 (25.00)	14 (16.87)	

Data are presented as mean ± SD for continuous variables and number (percentage) for categorical variables. Significant *p*-values (<0.05) are indicated in bold. Statistical comparisons were made using the Chi-square test (α) or independent *t*-test (β), as appropriate.

**Table 4:** Multivariable logistic regression of factors associated with short- and long-term complications.

Factors	Adjusted Odds Ratio	95% Confidence Interval	*p*-Value
**Short-term complications**			
EBL more than 5 mL/kg	54.131	2.190–1337.828	0.015
Serum albumin less than 3.5 g/dL	46.961	2.921–754.770	0.007
Serum hemoglobin less than 10 mg/dL	21.204	1.338–335.922	0.030
Patient with associated anomalies	17.312	1.096–273.433	0.043
Specimen weight	1.004	1.001–1.007	0.020
**Long-term complications**			
Patient presented with hypertension	18.706	1.717–191.164	0.018
Serum albumin less than 3.5 g/dL	7.417	1.1629–34.929	0.030

EBL: estimated blood loss.

**Table 5:** Survival probability by factors related to overall survival and progress-free survival.

Factors	n (%)	5-Year OS (%)	Log-Rank *p*-Value	5-Year PFS (%)	Log-Rank *p*-Value
**All**	83 (100)	82.43		68.08	
**Tumor stage**			0.41		0.09
- Stage I	13 (15.66)	90.00		67.50	
- Stage II	21 (25.30)	95.00		89.06	
- Stage III	34 (40.96)	73.26		56.80	
- Stage IV	8 (9.64)	83.33		85.71	
- Stage V (Bilateral)	7 (8.43)	75.00		28.57	
**Treatment protocol**			0.24		**0.03**
Primary nephrectomy	59 (71.00)	85.86		75.18	
Neoadjuvant chemotherapy	24 (28.92)	74.75		51.93	
**Tumor size**					
*Radiological size*			0.98		0.91
Radiological size < 10 cm	44 (53.01)	79.03		68.85	
Radiological size > 10 cm	39 (46.99)	85.78		67.12	
*Pathological size*			0.73		0.79
Pathological size < 10 cm	46 (55.42)	82.55		68.31	
Pathological size > 10 cm	37 (44.58)	82.64		67.37	
**Histologic subtype**			**<0.0001**		**0.005**
Favorable histology	73 (87.95)	88.45		75.30	
Unfavorable histology	10 (12.05)	50.00		30.00	
**SIOP histopathology risk**			**0.008**		**0.037**
Low risk	5 (6.02)	100.0		75.00	
Intermediate risk	64 (77.11)	86.89		75.81	
High risk	14 (16.87)	61.90		39.29	
**Histologic margin**			0.53		0.92
Negative	76 (91.57)	83.88		68.07	
Positive	7 (8.43)	68.57		71.43	
**Short-term complication**			**0.01**		0.15
Absent	69 (83.13)	87.43		71.17	
Present	14 (16.87)	58.20		51.43	
**Long-term complication**			0.55		0.95
Absent	71 (85.54)	80.57		68.67	
Present	12 (14.46)	90.91		64.81	

Significant *p*-values (<0.05) are indicated in bold.

## Data Availability

Data are contained within the article and Supplementary Materials.

## Supplementary Materials

The following supporting information can be downloaded at: https://www.mdpi.com/article/10.3390/curroncol32080413/s1, Supplementary Table S1: Correlation between short-term complications and pre-operative related factors, Supplementary Table S2: Correlation between short-term complications and operative and post-operative related factors, Supplementary Table S3: Chemotherapy and Radiotherapy for Wilms Tumor (SIOP Protocols), Supplementary Table S4: Radiotherapy in specific conditions (SIOP Protocols), Supplementary Table S5: Chemotherapy and Radiotherapy for Wilms Tumor (TPOG Protocols), Supplementary Table S6: Radiotherapy in specific condition (TPOG Protocols).
