# Supplementary material for: Predictors of Surgical Complications and Survival in Pediatric Wilms’ Tumor: A 20-Year Retrospective Study from Two Thai Centers
Source: Curr Oncol. 2025 Jul 23;32(8):413. doi: 10.3390/curroncol32080413 (PMC12384120; doi:10.3390/curroncol32080413)
Supplement: Supplementary file 1 [file curroncol-32-00413-s001.zip › curroncol-3730082-supplementary.pdf]

Appendix A

Supplementary Table S1. Correlation between short-term complications and pre-operative related factors

	No complication N=69	Complication N=14	*P-value*
Patient factors			
**Gender**			
Male	28 (40.58)	11 (78.57)	
Female	41 (59.42)	3 (21.43)	***0.009***$^{\alpha}$
**Age at surgery** (days)	978.65 (634.10)	877.35 (834.32)	*0.607*$^{\beta}$
**Body weight** (kg)	12.66 (4.10)	11.45 (3.82)	*0.617*$^{\beta}$
**Blood pressure (BP)**			
Systolic BP (mmHg)	113.73 (15.11)	114.92 (14.51)	*0.787*$^{\beta}$
Diastolic BP (mmHg)	71.27 (11.37)	74.92 (15.57)	*0.307*$^{\beta}$
**Associated anomaly**			
WAGR syndrome	4 (5.80)	4 (28.57)	***0.008***$^{\alpha}$
Horseshoe kidney	2 (2.90)	1 (7.14)	*0.438*$^{\alpha}$
**Pre-operative blood count**			
Hemoglobin (g/dL)	10.64 (1.64)	9.52 (2.15)	***0.031***$^{\beta}$
Hemoglobin < 10 g/dL	23 (33.33)	9 (64.29)	***0.030***$^{\alpha}$
Platelet (cell/dL)	389,130 (122,215)	347,428	*0.283*$^{\beta}$
WBC (cell/dL)	11,743 (3,979)	(173,373)	*0.833*$^{\beta}$
ANC (cell/dL)	5,970 (3,836)	11,491 (4552)	*0.741*$^{\beta}$
		5,601 (3,635)	
**Pre-operative renal**			
**function**	11.42 (4.41)	8.59 (6.06)	***0.043***$^{\beta}$
Blood urine nitrogen	0.38 (0.14)	0.35 (0.18)	*0.548*$^{\beta}$
(mg/dL)			
Creatinine (mg/dL)			
**Preoperative albumin**	4.07 (0.49)	3.67 (0.53)	***0.009***$^{\beta}$
**(g/dL)**	8 (12.70)	5 (38.46)	***0.025***$^{\alpha}$
Serum albumin < 3.5			
gm/dl			
**Pre-operative urinalysis**			
Pyuria	12 (17.39)	3 (21.43)	*0.720*$^{\alpha}$
Hematuria	24 (34.78)	4 (28.57)	*0.654*$^{\alpha}$
Tumor factors			
**Laterality of primary tumor**			
Left	38 (55.07)	8 (57.14)	
Right	28 (40.58)	2 (14.29)	
Bilateral	3 (4.35)	4 (28.57)	***0.006***$^{\alpha}$

Data are presented as mean ±SD for continuous variables and number (percentage) for categorical variables. Significant p-values (<0.05) are indicated in bold. Statistical comparisons were made using the Chi-square test (α) or independent t-test (β), as appropriate; WAGR syndrome: Wilms tumor, Aniridia, Genitourinary tract anomalies, Renal anomalies syndrome.

Supplementary Table S2. Correlation between short-term complications and operative and post-operative related factors

	No complication N=69	Complication N=14	*P-value*
Operative factors			
**Operative time** (mins)	279.63 (98.627)	317.28 (133.52)	0.224$^{\beta}$
**Estimated blood loss** (mL)	145.57 (341.38)	248.92 (278.67)	0.291$^{\beta}$
Estimated blood loss > 5 ml/kg	29 (42.03)	12 (85.71)	**0.003$^{\alpha}$**
**Resection status**			0.055$^{\alpha}$
Complete removal	65 (94.20)	11 (78.57)	
Incomplete removal	4 (5.80)	3 (21.43)	
**Post-operative staging**			
- Stage I	12 (17.39)	1 (7.14)	***0.041$^{\alpha}$***
- Stage II	18 (26.09)	3 (21.43)	
- Stage III	30 (43.48)	4 (28.57)	
- Stage IV	6 (8.70)	2 (14.29)	
- Stage V (Bilateral)	3 (4.35)	4 (28.57)	
**Pathological tumor size** (cm)	9.43 (2.42)	10.58 (8.43)	*0.145$^{\beta}$*
**Tumor size discrepancy** (cm)	1.52 (1.54)	1.64 (1.57)	*0.793$^{\beta}$*
**Tumor weight** (gm)	538.39 (281.93)	848.0 (491.61)	***0.004$^{\beta}$***
**Tumor volume** (cm^3)	939.09 (609.04)	1,524.51 (1,312.43)	***0.011$^{\beta}$***
**Local invasion**			
- Renal capsule	19 (27.54)	5 (35.71)	*0.538$^{\alpha}$*
- Renal sinus	14 (20.29)	4 (28.57)	*0.493$^{\alpha}$*
- Renal vein	6 (8.70)	2 (14.29)	*0.518$^{\alpha}$*
- Gerota 's fascia	4 (5.80)	1 (7.14)	*0.847$^{\alpha}$*
- Adjacent organ	4 (5.80)	0 (0)	*0.356$^{\alpha}$*
**Tumor rupture**	7 (14.29)	2 (14.29)	*0.678$^{\alpha}$*
**Tumor thrombus**	5 (7.69)	2 (15.38)	*0.376$^{\alpha}$*
**Paraaortic lymph node**	8 (11.59)	2 (14.29)	*0.778$^{\alpha}$*
**Intraabdominal ascites**	7 (10.61)	0 (0)	*0.202$^{\alpha}$*
Histologic subtype			*0.237$^{\alpha}$*
- Favorable histology	62 (89.86)	11 (78.57)	
- Unfavorable histology	7 (10.14)	3 (21.43)	
**Lymphovascular invasion**	28 (40.58)	7 (50.00)	*0.515$^{\alpha}$*
Post-operative outcomes			
**Time to initial feeding** (days)	2.17 (1.16)	3.43 (2.37)	***0.003$^{\beta}$***

	No complication N=69	Complication N=14	*P-value*
Time to full feeding (days)	3.56 (1.47)	6.21 (6.01)	***0.001$^{\beta}$***
Length of hospital stay (days)	13.51 (8.46)	23.28 (21.45)	***0.005$^{\beta}$***

Data are presented as mean ±SD for continuous variables and number (percentage) for categorical variables. Significant p-values (<0.05) are indicated in bold. Statistical comparisons were made using the Chi-square test (α) or independent t-test (β), as appropriate;

Supplementary Table S3. Chemotherapy and Radiotherapy for Wilms Tumor (SIOP Protocols)

Stage	Histology	Risk Group	Pre-op Chemotherapy	Post-op Chemotherapy	Radiotherapy
I	Intermediate/Favorable	Standard	VAD	VAD	None
I	High Risk (Blastemal type)	High	VAD	VAD + Doxorubicin + Cyclophosphamide	None
II	Intermediate/Favorable	Standard	VAD	VAD	None
II	High Risk (Blastemal type)	High	VAD	VAD + Doxorubicin + Cyclophosphamide	Consider flank RT if incomplete resection
III	Intermediate/Favorable	Standard	VAD	VAD + Doxorubicin	Flank RT (15 Gy)
III	High Risk (Blastemal/Anaplastic)	High	VAD	VAD + Doxorubicin + Cyclophosphamide	Flank RT (15–25.2 Gy depending on histology/margin)
IV	Any	High/Very High	VAD	HR Regimens	Flank RT
V	Any	Individualized	VAD	Based on final histology of each kidney	Depends on residual and local staging

VAD; Vincristine + Actinomycin D, RT; radiotherapy

Tumor Characteristics	Radiation Dose and field
Cytology-positive ascites or preoperative rupture or diffuse operative tumor spillage or peritoneal seeding	15 Gy Whole abdomen irradiation
Lung metastases	15 Gy whole lung irradiation (omit if complete response after chemo)
Brain metastases	30-36Gy whole brain irradiation
Liver metastases	25.2–30.6 Gy to focal metastasis site if unresectable

Bone metastases	30.6–40 Gy to the lesion plus 3 cm margin

Supplementary Table S4. Radiotherapy in specific conditions (SIOP Protocols)

Supplementary Table S5. Chemotherapy and Radiotherapy for Wilms Tumor (TPOG Protocols)

Stage	Histology	Risk Group	Chemotherapy (Regimen)	Radiotherapy
I	Favorable	Low	None or EE-4A	None
I	Favorable	Standard	EE-4A	None
I	Anaplasia	High	DD-4A	Flank RT (10.8 Gy)
II	Favorable	Standard	EE-4A	None
II	Focal anaplasia	High	DD-4A	Flank RT
II	Diffuse anaplasia	High	Regimen I	Flank RT
III	Favorable	Standard	DD-4A	Flank RT
III	Focal anaplasia	High	DD-4A	Flank RT
III	Diffuse anaplasia	High	Regimen I	Flank RT (19.8 Gy)
IV	Favorable	High	DD-4A	Flank RT and metastatic site
IV	Focal anaplasia	High	DD-4A	Flank RT and metastatic site
IV	Diffuse anaplasia	High	Regimen I	Flank RT (19.8 Gy) and metastatic site
V	Favorable	Individualized	Preop chemotherapy VAD for 6 wk, then tailored post-op	Based on residual disease/stage

EE-4A; Vincristine + Dactinomycin, DD-4A; Vincristine + Dactinomycin + Doxorubicin, Regimen I; Vincristine + Dactinomycin + Cyclophosphamide + Etoposide

Supplementary Table S6. Radiotherapy in specific condition (TPOG Protocols)

Tumor Characteristics	Radiation Dose and field

Cytology-positive ascites or preoperative rupture or diffuse operative tumor spillage or peritoneal seeding	10.8 Gy Whole abdomen irradiation
Lung metastases (Favorable histology)	12 Gy whole lung irradiation if no complete remission (metastatectomy first)
Lung metastases (Unfavorable histology)	12 Gy whole lung irradiation for all conditions (metastatectomy first)
Brain metastases	30.6Gy whole brain irradiation
Liver metastases	19.8 Gy to focal metastasis site if unresectable
Bone metastases	25.2 Gy to the lesion plus 3 cm margin
